# Hyperphosphatemia and Outcomes in Critically Ill Patients: A Systematic Review and Meta-Analysis

**DOI:** 10.3389/fmed.2022.870637

**Published:** 2022-05-17

**Authors:** Wen-He Zheng, Yan Yao, Hua Zhou, Yuan Xu, Hui-Bin Huang

**Affiliations:** ^1^Department of Critical Care Medicine, The Second People’s Hospital Affiliated to Fujian University of Traditional Chinese Medicine, Fuzhou, China; ^2^Department of Critical Care Medicine, Beijing Tsinghua Changgung Hospital, School of Clinical Medicine, Tsinghua University, Beijing, China

**Keywords:** hyperphosphatemia, critically ill, mortality, meta-analysis, prognosis

## Abstract

**Introduction:**

Serum phosphate level is often deranged during critical illness. Hyperphosphatemia, as a marker of disease severity, attracts more and more attention. This study aimed to evaluate the impact of hyperphosphatemia on clinical outcomes in critically ill patients.

**Methods:**

We searched for relevant studies in PubMed, EMBASE, and the Cochrane database up to Jan 10, 2022. Two authors independently screened studies, extracted data, and assessed the study quality. Meta-analyses were performed to determine hyperphosphatemia prevalence and evaluate its relationship with prognosis and important clinical outcomes. We also conducted subgroup analysis and sensitivity analyses to explore the sources of heterogeneity.

**Results:**

Ten studies with 60,358 patients met the inclusion criteria. These studies were moderate to high quality. The median prevalence of hyperphosphatemia was 30% (range from 5.6 to 45%). Patients with hyperphosphatemia had a significantly higher risk of all-cause mortality than those without (OR 2.85; 95% CI, 2.35 to 3.38, *P* < 0.0001). Subgroup analyses, sensitivity analyses, and regression analyses further confirmed these results. In addition, patients with hyperphosphatemia required more CRRT (OR 4.96; 95% CI, 2.43 to 10.2, *P* < 0.0001) but not significantly increased duration of mechanical ventilation (mean difference, MD 0.13, 95% CI −0.04 to 0.30; *P* = 0.138), length of stay in intensive care unit (ICU) (SMD 0.164 day, 95% CI −0.007 to 0.335; *P* = 0.06), and length of stay in hospital (SMD 0.005 day, 95% CI −0.74 to 0.75; *P* = 0.99).

**Conclusions:**

Our results indicated that hyperphosphatemia was associated with all-cause mortality in critically ill patients. However, due to the retrospective design of the included studies, more prospective, well-designed research is required in the future.

**Systematic Review Registration:**

[https://doi.org/10.37766/inplasy2021.12.0130], identifier [INPLASY2021120130].

## Introduction

Serum phosphorus disorders, including hypophosphatemia and hyperphosphatemia, are common findings in critical illness (1, 2). The incidence of acute blood phosphorus abnormalities admitted to the intensive care unit (ICU) can be as high as 45% (3). Many endogenous and exogenous factors can lead to serum phosphorus dyshomeostasis, such as intestinal loss, sepsis, trauma, impaired renal clearance, catabolic processes, diabetic ketoacidosis, acid-base disorders, and many medications (1, 4, 5). In total, 30% of serum phosphate exists in inorganic form and constitutes the clinically measured component of blood phosphorus with a normal reference range of 0.7–1.5 mmoL/L (6). Despite low levels in the body, serum phosphorus plays a vital role in many physiological produces such as energy metabolism, bone metabolism, and cell signaling and affects almost all organ systems (7). Therefore, it is crucial to maintain normal blood phosphorus levels.

Compare to hypophosphatemia that has been attracted more clinical attention (1), acute hyperphosphatemia in the ICU setting seems to be often overlooked, which might relate to its usually mild symptoms, mainly limited to concomitant hypocalcemia (e.g., muscle weakness, hand and foot twitching, cardiac arrhythmias, and hypotension). Meanwhile, treatment of hyperphosphatemia requires identification and correction of the underlying cause, with the therapy goals of normalizing serum phosphorus concentrations (2.7–4.5 mg/dL), avoiding or resolving symptoms of hyperphosphatemia, and maintaining serum (calcium × phosphorus) at less than 55–60 mg^2^/dL^2^ (8). On the other hand, the importance of hyperphosphatemia has been largely and consistently emphasized in patients with chronic kidney disease (CKD) and chronic cardiovascular disease (9, 10); however, studies on its prognosis in critically ill patients have come to different conclusions. Thus, it remains unclear whether hyperphosphatemia correlates with prognosis or is merely a marker of severe disease.

Several studies on hyperphosphatemia in critical ill patients have been published recently, though some have a modest sample size (4, 9, 11, 12). Therefore, with the power of meta-analysis, we aimed to perform a systematic review and meta-analysis to explore the prognostic value of hyperphosphatemia in such a patient population.

## Methods

We followed the PRISMA guidance to prepare the present systematic review and meta-analysis (13) (Additional File 1). The protocol for this study has been registered on the International Platform of Registered Systematic Review and Meta-analysis Protocols database (INPLASY2021120130) and is available in full on inplasy.com (https://inplasy.com/inplasy-2021-12-0130/).

### Search Strategy and Selection Criteria

Two authors (W-HZ and YY) independently systemically searched PubMed, EMBASE, and Cochrane Library from inception through Jan 10, 2022 (the last search date). Search terms included “hypophosphatemia,” AND “critical care,” “critically ill,” “intensive care,” using a combination of the Medical Subject Headings and keywords. Details of the complete search strategy are attached in Additional File 2. The search included all study designs with no language restriction. We imported the studies into Endnote to exclude duplicate studies and then screened literature (titles, abstracts, and full texts) for relevant articles. We also reviewed the reference lists of included full-text articles for more potential studies. Disagreements were solved by discussions between the two authors or decided by a third author (H-BH).

We considered including the studies if they met the following PICOS criteria: (1) Patients: adult (>18 years old) critically ill patients; (2) Exposure: hyperphosphatemia (defined by the authors); (3) Comparator: normal phosphate level; and (4) Outcomes: mortality and other clinical outcomes; (5) Study design: cohort, case-control or randomized controlled design. We excluded the studies that did not report clear hyperphosphatemia definitions, provide prognostic outcomes, or studies recruited children or pregnant women. Studies available only in comment, abstract or meeting reports were also excluded. We contacted the authors if the data on predefined outcomes from their studies were required.

### Data Extraction and Quality Assessment

Two reviewers (W-HZ and YY) independently extracted data from the included studies, such as study design, country, publication year, sample size, setting, follow up and severity of illness, outcomes of interest, hyperphosphatemia criteria and prevalence, and methodological quality.

The above two independent reviewers (W-HZ and YY) evaluated the quality of each included study using the Newcastle-Ottawa Scale (NOS) for cohort studies (14). The NOS has three domains based on the cohort’s selection, the groups’ comparability, and the outcomes’ quality. A study was given a maximum of one point in each item within the Selection and Outcome domains and a maximum of two points for the Comparability domain. The scale scores ranged from 0 to 9 points, with 8 or 9 classified as high quality, 6 or 7 as moderate quality, and five or below as low quality. Discrepancies were identified and resolved through discussion.

### Outcomes

The primary outcome was the short-term mortality rate (ICU or hospital mortality or mortality within a 90-day follow-up after admission, the longest observation period was preferred). Secondary outcomes were the duration of mechanical ventilation (MV), length of stay in the hospital (LOS), and CRRT requirement.

### Statistical Analysis

We combined the results from all relevant studies to estimate the pooled odds ratio (OR) and associated 95% confidence intervals (CIs) for dichotomous outcomes and to estimate mean differences (MD) and 95% CI as the effective results for continuous outcomes. Before data analysis, we estimated the mean from median and standard deviations (SD) from IQR using the previous study’s methods, if require (15). According to the different reporting forms of mortality provided by the included studies, we separately conducted two types of meta-analyses for the risk estimation between hyperphosphatemia and all-cause mortality as follows: (1) For studies reporting mortality rates (crude data) between patients with or without hyperphosphatemia, we calculated OR and associated 95% CI as the primary outcome. (2) For studies utilizing regression analyses to adjust for effects of confounding factors on mortality, we combine the mortality estimates with corresponding standard errors by the generic inverse variance method in the sensitivity analysis. Thus, the OR and hazard ratio (HR) reported in these studies required natural logarithmic transformations before merging. If not stated otherwise, we preferred adjusted analysis results.

We used the *I*^2^ statistic to examine the heterogeneity across these trials. An *I*^2^ > 50% indicates significant heterogeneity. We chose a fixed-effect model for *I*^2^ < 50% and a random-effect model for *I*^2^ ≥ 50%. We assessed publication bias by visually exploring funnel plots for asymmetry, Egger’s test, and Begg’s test. In all analyses, we used STATA version 14.0 (STATA Corporation, College Station, TX, United States).

### Additional Analyses

To explore the potential influence factors for the primary outcome, we performed subgroup analyses by pooling studies with the following properties: (1) Geographic location: Asian or not Asian countries; (2) Sample size:>200 or ≤200; (3) Measured unit: >4.5 mg/dL or >1.5 mmol/L; (4) Follow-up duration: long-term (mortality at least 6 months follow-up after hospital discharge) or short-term; (5) Using initial serum phosphate level or not, and (6) Mortality prevalence: mortality rate <30%, between 30 and 45%, or >45%. Additionally, we conducted sensitivity analyses by excluding one study at a time to explore whether an individual study’s particular result drove the results. We also conducted sensitivity analyses by pooling studies that only reported 30-, 60-, and 90-day, hospital discharge, ICU mortality, or utilizing regression analyses to investigate the relationship between hyperphosphatemia and mortality.

## Results

### Study Selection

**FIGURE 1:**
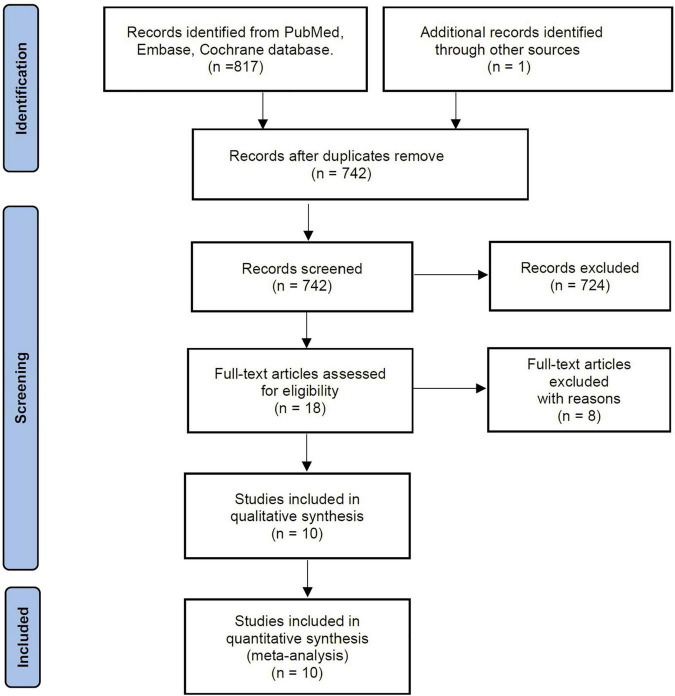
Selection process for the studies included in the meta-analysis.

Our search identified 817 records from defined databases. After screening the titles and abstracts, 18 were qualified for full-text review. Based on the full-text evaluation, we excluded 8 studies summarized in Additional File 3 (Supplementary Table 3) with exclusion reasons based on the full-text evaluation. Thus, the remaining 10 studies (4, 9, 11, 12, 16–21) were included in the final meta-analysis (Figure 1).

### Study Characteristics and Methodological Quality

The main characteristics of the included studies concerning 60,358 adult patients are summarized in Table 1, and the predefined outcome details are provided in Table 2. These studies were retrospective designs and were published between 2013 and 2021 from 7 countries (Austria *n* = 3, China *n* = 3, Korea *n* = 1, United States *n* = 1, Sweden *n* = 1, and Saudi Arabia *n* = 1). Included studies focused on patients from ICU (4, 9, 11, 12, 17, 18, 20, 22), trauma centers (21), or severe burn (19), with sample sizes ranging from 197 to 32,333. On average, the mean age of patients was 59.5 years, and 60% of them were male. The mortality endpoints varied among the included studies. The 8 of the 10 included studies reported the prevalence of hyperphosphatemia, with the mean prevalence ranging from 5.6–45%. When calculated, the mean prevalence was 21% (11,397/54,012). Nine studies provided mortality rates between hyperphosphatemia and normal serum phosphorus, we investigated mortality between groups using OR and associated 95% CI as the primary outcome. Meanwhile, nine studies utilized regression analyses to adjust for effects of confounding factors on mortality was pooled and investigated using sensitivity analysis. In addition, a total of nine included studies (4, 9, 11, 12, 16, 17, 19–21) provided data on hypophosphatemia in the prognosis and important clinical outcomes, and the main conclusions were summarized in Additional File 5. Overall, almost all the included studies showed no association between mortality and baseline hypophosphatemia.

**TABLE 1:** Characteristics of included studies in the current meta-analysis.

Study	Country	Design	Cohort	Definition	Sample size	Age, year	Male,%	Follow up	Mortality*^a^*, % (HPP/NP)	NOS
Broman (11)	Sweden	R	General ICU	>1.5 mmol/L	4143	62.3	41.6	180 days	47.7/20.1	8
Chen (16)	China	R	ICU patients	>4.5 mg/dL	32333	64	56	Hospital LOS	31.3/16.4	8
Al Harbi (17)	Saudi Arabia	R	General ICU	>4.5 mg/dL	1422	66.7	58.8	Hospital LOS	44.7/26.1	8
Jung (9)	Korea	R	Patients with CRRT	>4.5 mg/dL	1144	63.2	61.6	90 days	73.9/65.7	8
Miller (19)	United States	R	General ICU	>4.2 mg/dL	197	60.1	47.2	28 days	58.5/32.5	7
Rugg (20)	Austria	R	Polytrauma patients	>1.5 mmol/L	166	47	77.7	Hospital LOS	35.7/5.5	8
Sin (4)	Austria	R	General ICU	>1.5 mmol/L	13155	60	56	30 days	15.6/5.5	8
Wang (12)	China	R	Sepsis patients	>4.5 mg/dL	4767	65.2	55.7	Hospital LOS	37.1/21.6	8
Kuo (18)	China	R	Burn patients	>4.5 mg/dL	301	45.2	77.8	90 days	51.9/14.1	8
Suzuki (21)	Austria	R	General ICU	>1.4 mmol/L	2730	61.3	60.4	Hospital LOS	22.5/16.4	8

*^a^Defined as mortality rate of longest follow-up. CRRT, continuous renal replacement therapy; ED, emergency department; HPP, hyperphosphatemia; LOS, length of stay; OS, Newcastle-Ottawa Scale; NP, normal phosphate; ICU, intensive care unit; R, retrospective.*

**TABLE 2:** Predefined outcomes of included studies.

Study/year	ICU mortality,%		Hospital mortality,%		90-day mortality,%		60-day mortality,%		30-day mortality,%	
	Hyper-P	Control	Hyper-P	Control	Hyper-P	Control	Hyper-P	Control	Hyper-P	Control
Broman (11)					535/1215 (44)	338/2002 (16.9)	517/1215 (42.6)	310/2002 (15.5)	476/1215 (39.2)	271/2002 (13.5)
Chen (16)	839/5031 (16.7)	1144/20184 (5.7)			1573/5031 (31.3)	3119/19040 (16.4)			1228/5031 (24.4)	2175/19040 (11.4)
Al Harbi (17)	114/369 (30.9)	143/865 (16.5)	165/369 (44.7)	226/865 (26.1)						
Jung (9)										
Miller (19)									24/41 (58.5)	40/123 (32.5)
Rugg (20)	18/56 (32.1)	5/120 (4.2)	20/56 (35.7)	6/120 (5.0)						
Sin (4)									396/2544 (15.6)	504/9187 (5.5)
Wang (12)			224/829 (27)	391/3107 (12.6)	315/849 (37.1)	672/3107 (21.6)	298/849 (35.1)	617/3107 (19.9)		
Kuo (18)					28/52 (53.8)	45/248 (18.1)	28/52 (53.8)	42/248 (16.9)	28/52 (53.8)	42/249
Suzuki (21)	209/1240 (16.9)	119/1490 (8.0)	369/1240 (29.8)	245/1490 (16.4)						
Broman (11)										
Chen (16)					2.62 (1.38. 5.16)		95/5031 (1.9)	32/19040 (0.2)		
Al Harbi (17)	2 (0, 6)	1 (0, 6)	17 (7.39)	22 (12.51)	2.8 (0.7,1.0)	3.9 (1.04, 10.04)	78/369 (21.1)	64/865 (7.4)		
Jung (9)										
Miller (19)	3.0 (1.8, 6.8)	4.8 (2.3, 10.5)	10 (5, 16.6)	15.5 (10, 22.8)	5.0 (2.3, 9.5)	7.8 (4.8, 13.5)	19/41 (46.3)	21/83 (25.3)		
Rugg (20)			12 (4, 27)	21 (11, 34)	7 (3, 12)	8 (2, 14)				
Sin (4)					3 (2, 5)	2 (2, 4)	369/2544 (14.5)	100/9187 (10.9)		
Wang (12)			12.4 (11.2)	10.6 (9.9)	6.2 (7.1)	5.0 (5.9)				
Kuo (18)							8/52 (15.4)	20/249 (8.0)		
Suzuki (21)	19 (0, 111.8) h	7 (0, 38) h	15.0 (7.0, 32.3)	12.1 (6.6, 25.5)	3.6 (1.7, 8.0)	2.0 (0.95–3.9)	154/1340 (11.5)	52/1490 (3,5)		

*Results were expressed as median (1st quartile, 3rd quartile), mean (standard deviation), or number (%). Hyper-P, hyperphosphatemia; LOS, length of stay; CRRT, continuous renal replacement therapy; ICU, intensive care unit.*

The quality assessment is available in Additional File 6 and shows the study quality ranging from moderate to high quality, with scores ranging from 7 to 9 on the NOS scale. Nine studies were considered high quality, and the other two studies were of moderate quality.

### Primary Outcome

**FIGURE 2:**
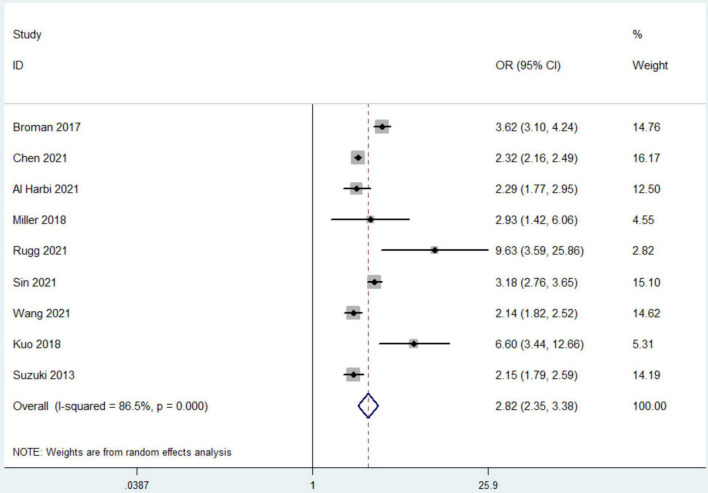
The forest plot in assessing the impact of hyperphosphatemia on mortality. CI, confidence interval; OR, odds ratio. Pooled analyses from crude data provided by associated included studies.

All included studies reported the outcome of all-cause mortality. Nine studies with 47,570 patients compared mortality rates between patients with or without hyperphosphatemia (4, 11, 12, 16–21). Among these patients, 11,504 had hyperphosphatemia, and 3,469 died (30.2%) compared to 36,173 patients of normal serum phosphorus level with 5,250 deaths (14.5%) observed. Hyperphosphatemia was associated with a higher risk of mortality (OR 2.82, 95% CI 2.35 to 3.38, random effect model) with the heterogeneity of 86.5% observed (Figure 2). We found no evidence of publication bias with Begg’s test or Egger’s test (*P* = 0.443), and the funnel plots did not suggest asymmetry (Additional File 7).

Subsequently, we conducted subgroup analyses to explore potential heterogeneity sources. In terms of between groups mortality analyses, hyperphosphatemia was associated with a higher risk of mortality in all the subgroups, including sample size, geographic location, using initial serum phosphate level or not, measured unit, duration of follow-up, or mortality prevalence (all *P*-values < 0.0001 with *I*^2^ ranging from 0 to 90.9%) (Table 3).

**TABLE 3:** Subgroup analysis and sensitivity analyses on risk of mortality in patients with hyperphosphatemia.

		References	Patient number	Odds ratio (95% CI)	*I* ^2^	*p*
**Subgroup analysis:**						
Sample size	>200	(4, 11, 12, 16–18, 21)	47,240	2.70 [2.26, 3.25]	88.5%	<0.0001
	<200	(19, 20)	330	5.06 [1.58, 16.2]	72.4%	<0.0001
Geographic location	Asian	(12, 16–18)	29.562	2.42 [2.00, 2.92]	83.4%	<0.0001
	Not Asian	(4, 11, 19–21)	18,008	3.68 [2.00, 4.51]	72.5%	<0.0001
Measurement unit	>4.2 mg/dL	(12, 16–19)	27,570	2.44 [2.05, 2.90]	87.5%	<0.0001
	>1.5 mmol/L	(4, 11, 20, 21)	17,844	3.19 [2.37, 4.29]	64.6%	<0.0001
Follow-up	≤30 days	(4, 19)	1,2032	3.16 [2.76, 3.63]	0%	<0.0001
	Hospital LOS	(12, 16, 17, 20, 21)	32,157	2.28 [2.01, 2.59]	57.4%	<0.0001
	≥90 days	(11, 19)	3,381	4.49 [2.55, 7.88]	67.6%	<0.0001
As initial values	Not	(19, 21)	2,894	2.19 [1.84, 2.62]	0%	<0.0001
	Yes	(4, 11, 12, 16–18, 20)	44,676	2.98 [2.40, 3.69]	89.2%	<0.0001
Mortality prevalence	<30%	(4, 21)	14,461	2.63 [1.79, 3.85]	90.9%	<0.0001
	30–45%	(11, 12, 16, 17, 20)	32,644	2.71 [2.13, 3.46]	89%	<0.0001
	>45%	(18, 19)	465	4.47 [2.01, 9.94]	62.8%	<0.0001
**Sensitivity analysis**						
Risk of mortality	30-day	(12, 17, 20, 21)	8,086	3.36 [2.59, 4.35]	88.5%	<0.0001
	60-day	(11, 12, 18)	7,474	3.50 [2.06, 5.94]	93.4%	<0.0001
	90-day	(11, 12, 16, 18)	31,545	2.92 [2.15, 3.97]	92.6%	<0.0001
	Hospital	(12, 17, 20, 21)	8,086	2.47 [1.96, 3.11]	67.1%	<0.0001
	ICU	(16, 17, 20, 21)	28,201	2.93 [2.13, 4.04]	83.2%	<0.0001
	>4.5 mg/dL	(4, 11, 12, 16–18, 20)	44,676	3.12 [2.52, 3.86]	88.3%	<0.0001
	>4.2 mg/dL	(4, 11, 12, 16–21)	47,570	3.10 [2.53, 3.81]	85.5%	<0.0001
	Adjusted OR*	(4, 12, 17, 18, 20)	21,718	1.50 [1.29, 1.75]	21.5%	<0.0001
	Adjusted HR*	(9, 11, 16, 18)	28,733	1.59 [1.03, 2.14]	96.5%	<0.0001

HR, hazard ratio; ICU, intensive care unit; LOS, length of stay; OR, odds ratio. *OR/HR were adjusted by predefined variables (i.e., Age, gender, ethnicities, vital sign, disease severity, or treatments) by authors.

In the sensitivity analyses, hyperphosphatemia were associated with significantly increased mortality when compared with normal serum phosphorus at hospital discharge, ICU duration, 30-, 60-, and 90-day after recruitment, with the OR of 2.47 (95% CI 1.96, 3.11), 2.93 (95% CI 2.13, 4.04), 3.36 (95% CI 2.59, 4.35), 3.50 (95% CI 2.06, 5.94), and 2.92 (95% CI 2.15, 3.97), respectively. As to the nine studies utilizing regression analyses, five with multivariate logistic regression analyses (4, 12, 17, 18, 20) and four with Cox proportional hazard regression analyses (9, 11, 16, 18) were pooled for assessing the risk for mortality. Pooled data showed hyperphosphatemia was associated with an increased risk of mortality (adjusted OR 1.50, 95% CI 1.29–1.75, *P* < 0.0001, and adjusted HR 1.59, 95% CI 1.03–2.14, *P* < 0.00001), respectively (Table 3).

### Secondary Outcomes

**FIGURE 3:**
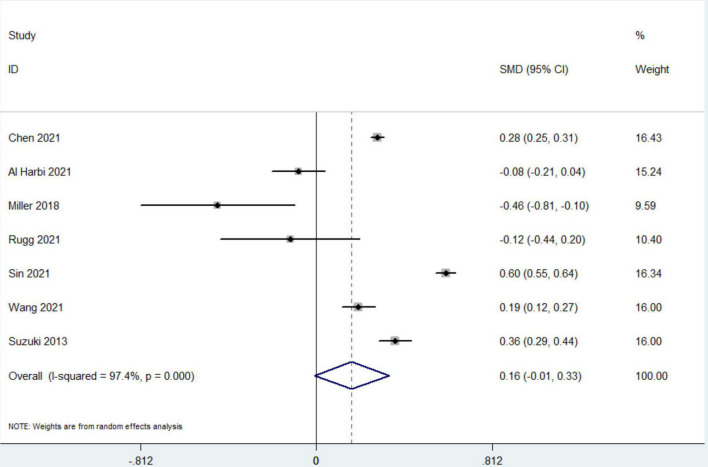
The forest plot in assessing the impact of hyperphosphatemia on length of stay in ICU. CI, confidence interval, SMD, standardized mean difference. Pooled analyses from crude data provided by associated included studies.

**FIGURE 4:**
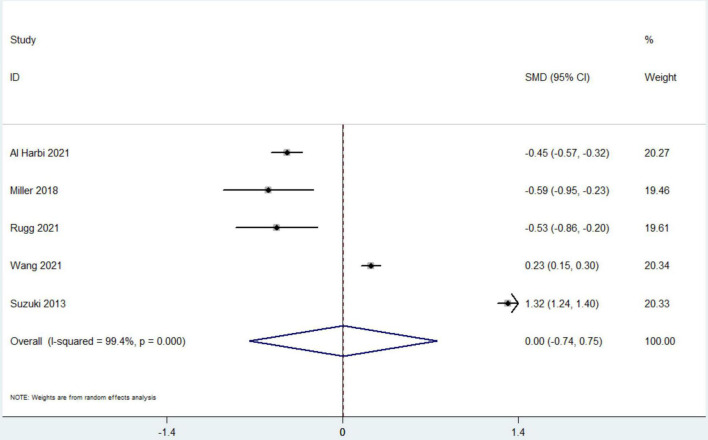
The forest plot in assessing the impact of hyperphosphatemia on length of stay in hospital. CI, confidence interval; SMD, standardized mean difference. P Pooled analyses from crude data provided by associated included studies.

**FIGURE 5:**
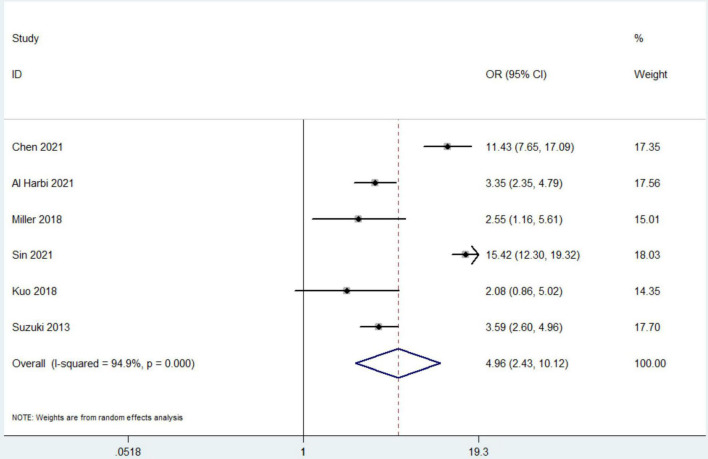
The forest plot in assessing the impact of hyperphosphatemia on risk of receiving continuous renal replacement therapy. CI, confidence interval, OR, odds ratio. Pooled analyses from crude data provided by associated included studies.

**FIGURE 6:**
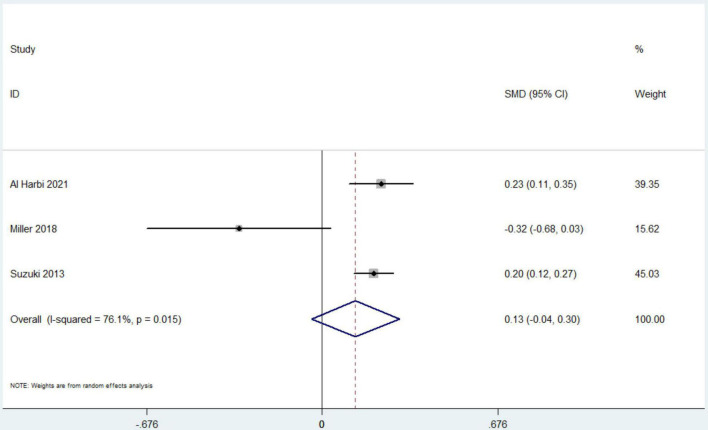
The forest plot in assessing the impact of hyperphosphatemia on duration of mechanical ventilation. CI, confidence interval; SMD, standardized mean difference. Pooled analyses from crude data provided by associated included studies.

Data from 7 studies indicated that hyperphosphatemia was not associated with decreased ICU LOS (*n* = 44,052; SMD 0.16 day, 95% CI −0.01 to 0.33; *I*^2^ = 97.4%; *P* = 0.06) (4, 12, 16, 17, 19–21) (Figure 3). Five studies reported hospital LOS as an interest (12, 17, 19–21). Pooled the analysis showed that hyperphosphatemia did not significantly increase hospital LOS compared to normal serum phosphorus (*n* = 8,250; SMD 0.005 day, 95% CI −0.74 to 0.75; *I*^2^ = 99.4%; *P* = 0.99) (Figure 4). CRRT requirement during the follow-up was available from 6 studies (4, 16–19, 21) and indicated significantly increase in the hyperphosphatemia group (*n* = 40,291; OR 4.96, 98% CI 2.43 to 10.12; *I*^2^ = 94.9%; *P* < 0.0001) (Figure 5). In addition, the duration of MV was reported by 3 studies and was comparable between the two groups with or without hyperphosphatemia (*n* = 4,128; SMD 0.13, 98% CI−0.04 to 0.30; *I*^2^ = 76.1%; *P* = 0.138) (17, 19, 21) (Figure 6).

## Discussion

To the best of our knowledge, the current study is the first systematic review and meta-analysis investigating the impact of hyperphosphatemia on clinical outcomes. Our results showed that critically ill patients usually suffer hyperphosphatemia, with the prevalence ranging from 5.6 to 67.9% (4, 9, 11, 12, 16–21). Hyperphosphatemia was an independent risk factor of short-term mortality by a nearly twofold increase. Further subgroup analyses and sensitivity analyses confirmed this finding. Moreover, hyperphosphatemia was associated with a significantly increased CRRT requirement, but not the duration of MV, ICU and hospital LOS.

Our study has several strengths. The current meta-analysis provided robust evidence to fill the gaps in previous guidelines (22); that is, hyperphosphatemia can be evaluated to assess prognosis in critically ill patients. Second, our findings are consistent with the previous guideline of other patient populations, including CKD, cardiovascular disease with normal renal function, and tumor lysis syndrome, highlighting adverse clinical outcomes with hyperphosphatemia (10, 23). As a result, our study adds a new population of evidence. Third, we have thoroughly evaluated mortality risk from several general ICU and trauma/burn center cohorts, including mortality between hyperphosphatemia with normal levels and linear relationships between hyperphosphatemia and mortality. In addition, we included a large sample size of more than 60,000 cases, with sufficient statistical power to allow for subgroup analyses and sensitivity analyses based on different potential influencing factors. The results were reliable and further supported the robustness of our main results.

Our results showed that the incidence of hyperphosphatemia in the included studies was approximately 21% (11,397/54,012, 9 studies), but the incidence varied widely between studies (17–45%). This might be related to the different ICU populations selected by the included studies. As in several studies recruiting non-selected ICU patients, the hyperphosphatemia incidence ranged from 18.5 to 26.3% (4, 11, 16, 19, 21). In one study focusing only on patients requiring CRRT, hyperphosphatemia was found in 721 (67.5%) patients before starting CRRT and remained in 399 (40.6%) at 24 h after CRRT initiation (9). For polytrauma patients, the incidence was about 33.7% (21). However, it is note that all these studies retrospectively included only patients with serum phosphate levels measured, so these reported incidence rates may be underestimated.

Compared with a bulk of literature focusing on the origins and consequences of hyperphosphatemia in chronic renal and cardiovascular disease in non-ICU patients (10), the causes of hyperphosphatemia in ICU patients are much more complex. Besides decreased renal clearance or too large an intake (e.g., phosphate-containing laxatives), ischemic tissue injury, such as intestinal or critical limb ischemia, coronary heart disease, cardiac arrest, lactic acidosis and diabetic ketoacidosis may cause hyperphosphatemia (24–29). However, we could not further evaluate the association between the causes and mortality in the present meta-analysis because specific data from included studies were not available. Also, patients may have multiple origins of hyperphosphatemia and have undergone different therapeutic management to restore normal blood phosphorus concentrations (4, 9, 11, 12, 16–21).

Our results showed a lower survival rate in patients with hyperphosphatemia, with a mean mortality rate of approximately 30.4% (15–58.5%) (4, 9, 11, 12, 16–21). However, the relevance of hyperphosphatemia to prognosis needs further discussion. First, most included studies assessed baseline blood phosphorus concentrations at ICU admission, rather than described overall phosphate exposure to describe the effect of hyperphosphatemia on ICU patients. Only one included study added to this gap (19). In this retrospective cohort study of 197 mechanically ventilated patients with severe sepsis or septic shock, Miller and colleagues found that time-weighted hypophosphite patients were more likely to die within 28 days of admission to the ICU (19). More studies are needed in the future to focus on patients’ phosphate levels throughout their ICU stay, which may better assess the impact of phosphate status on the prognosis. Second, hyperphosphatemia appears to be associated with mortality in a dose-dependent manner. In a study focusing on sepsis patients with abnormal initial phosphate concentration, the authors suggested that moderate to severe hyperphosphatemia, but not mild hyperphosphatemia, was an independent prognostic factor of mortality (30). This appears to partly explain the variations in mortality rates among the ICU hyperphosphatemic patients.

Third, the prognosis of hyperphosphatemia might be related to its treatment responsiveness. In the study by Kuo et al. (18), the authors reported that patients with severe burns and initial hyperphosphatemia had lower 90-day survival. Furthermore, they found the worst survival among patients with persistently high phosphate levels among patients who survived longer than 3 days. Similar results were also found in the study by Jung and colleagues (9), who demonstrated that patients with increased phosphate levels during 24 h were at a higher risk of death than those with stable (*P* = 0.001) or decreased phosphate levels (*P* < 0.001). These results suggested that timely correction of hyperphosphatemia may be a necessary therapeutic measure. On the other hand, mortality rates were higher in patients without improvement after treatment of hyperphosphatemia, again suggesting that residual hyperphosphatemia still had prognostic value (9).

Interestingly, most included studies (9/10) concluded that hypophosphatemia did not predict prognosis (4, 9, 11, 12, 16, 17, 19–21 (Appendix File 4). However, as another phosphorus disorder, hyperphosphatemia is an independent prognostic predictor, not just a marker of severity. Thus, it should deserve more clinical attention. In addition, in several studies reporting a higher CRRT requirement in the hyperphosphatemic group (4, 12, 16–18, 20, 21), there was clear evidence of more renal injury in the hyperphosphatemic group, including more renal failure patients (12, 16–18) or higher creatinine levels (4, 21). This evidence supports the idea that renal failure is a significant cause of hyperphosphatemia. Phosphate homeostasis is strongly dependent on adequate renal excretion. Thus, from this perspective, more CRRT requirements is likely to reflect renal failure as the cause of hyperphosphatemia rather than hyperphosphatemia *per se*. Also, all the included studies did not provide indications for the use of CRRT in hyperphosphatemia and its therapeutic effects. Therefore, we could not draw an association between phosphate levels and CRRT requirements based on the results of the current meta-analysis.

Several limitations must be considered in our meta-analysis. First, the observational design of all included studies excluded any causal inference. Meanwhile, only patients who underwent serum phosphorus testing were included in retrospective studies, which is prone to selection bias. Second, most studies assessed only baseline serum phosphate levels at ICU admission, ignoring assessments of serum phosphate levels over time. Third, most studies lack data on levels of parathyroid hormone, FGF23, and 1,25 -dihydroxyvitamin D3. Fourth, in addition to traumatic populations, most studies focus on unselected critically ill patients, and the uneven distribution of different underlying diseases in these studies may also exert different prognostic values. Fifth, in the subgroup and sensitivity analyses, we could not have considered all the confounding factors that might play a role in linking phosphate levels to ICU mortality, such as the effects of CRRT intensity, phosphatidic hormone levels, nutritional status, and artificial nutrition. Sixth, only a few studies suggest the severity of phosphate abnormalities and their prognostic impact, but the lack of a grading scale prevents further studies. Finally, whether correction of hyperphosphatemia could reduce mortality in ICU patients was still required further large-sample, prospective studies to confirm.

## Conclusion

The hyperphosphatemia is commonly seen and varied in ICU patients. Hyperphosphatemia is a reliable factor for overall mortality in such a patient population. These findings were further confirmed in subgroup and sensitivity analysis. However, due to the retrospective design of the included studies, more prospective, well-designed research is required in the future.

## Data Availability Statement

The original contributions presented in the study are included in the article/Supplementary Material, further inquiries can be directed to the corresponding author.

## Author Contributions

H-BH contributed to the conception of the study, analysis and drafting of the manuscript, and were responsible for the integrity of the work as a whole and from inception to publication of the manuscript. W-HZ, YY, and HZ contributed to data collection and analysis. YX contributed to design and revisions of this manuscript. All authors contributed to the article and approved the submitted version.

## Conflict of Interest

The authors declare that the research was conducted in the absence of any commercial or financial relationships that could be construed as a potential conflict of interest.

## Publisher’s Note

All claims expressed in this article are solely those of the authors and do not necessarily represent those of their affiliated organizations, or those of the publisher, the editors and the reviewers. Any product that may be evaluated in this article, or claim that may be made by its manufacturer, is not guaranteed or endorsed by the publisher.
