# Supplementary material for: Hyperphosphatemia and Outcomes in Critically Ill Patients: A Systematic Review and Meta-Analysis
Source: Front Med (Lausanne). 2022 May 17;9:870637. doi: 10.3389/fmed.2022.870637 (PMC9156794; doi:10.3389/fmed.2022.870637)
Supplement: Supplementary file 1 [file Data_Sheet_1.docx]

**Hyperphosphatemia and Outcomes in Critically Ill patients: A Systematic Review and Meta-Analysis**

**Wen-He Zheng^1^, MD; Yan Yao^2^, MD; Yuan Xu^2^, MD; Hui-Bin Huang^2*^ MD**

***Corresponding author:**

**Hui-Bin Huang,** Email: [hhba02922@btch.edu.cn](mailto:hhba02922@btch.edu.cn).

**Additional file**

Additional file 1

Checklist following PRISMA guideline………………………………………………………………………………………………………..1

Additional file 2

Search strategy………………………………………………………………………………………………….….……………………..…..………..5

Additional file 3

Table S3: Studies needed for full-reviewed but not included in the current meta-analysis………….……………..7

Additional file 4

The predefined outcomes…………………………………………………………………………..…………………………….…….….......…8

Additional file 5

Summarizing finding of included studies for the hypophosphatemia and prognosis in ICU patients……...10

Additional file 6

Table S6: Quality assessment and overall risk of bias of included studies………………….…………………………......11

Additional file 7

Figure S7: Publication bias………………………………………..……………………………………………………….….….……………14

**Additional file 1**

**PRISMA 2009 checklist**

| **Section/topic** | **#** | **Checklist item** | **Reported on page #** |
| --- | --- | --- | --- |
| **TITLE** | | |  |
| Title | 1 | Identify the report as a systematic review, meta-analysis, or both. | 1 |
| **ABSTRACT** | | |  |
| Structured summary | 2 | Provide a structured summary including, as applicable: background; objectives; data sources; study eligibility criteria, participants, and interventions; study appraisal and synthesis methods; results; limitations; conclusions and implications of key findings; systematic review registration number. | 2 |
| **INTRODUCTION** | | |  |
| Rationale | 3 | Describe the rationale for the review in the context of what is already known. | 4 |
| Objectives | 4 | Provide an explicit statement of questions being addressed with reference to participants, interventions, comparisons, outcomes, and study design (PICOS). | 5 |
| **METHODS** | | |  |
| Protocol and registration | 5 | Indicate if a review protocol exists, if and where it can be accessed (e.g., Web address), and, if available, provide registration information including registration number. | 6 |
| Eligibility criteria | 6 | Specify study characteristics (e.g., PICOS, length of follow-up) and report characteristics (e.g., years considered, language, publication status) used as criteria for eligibility, giving rationale. | 6 |
| Information sources | 7 | Describe all information sources (e.g., databases with dates of coverage, contact with study authors to identify additional studies) in the search and date last searched. | 6 |
| Search | 8 | Present full electronic search strategy for at least one database, including any limits used, such that it could be repeated. | 6 and Appendix file 2 |
| Study selection | 9 | State the process for selecting studies (i.e., screening, eligibility, included in systematic review, and, if applicable, included in the meta-analysis). | 6 |
| Data collection process | 10 | Describe method of data extraction from reports (e.g., piloted forms, independently, in duplicate) and any processes for obtaining and confirming data from investigators. | 6-7 |
| Data items | 11 | List and define all variables for which data were sought (e.g., PICOS, funding sources) and any assumptions and simplifications made. | 7 |
| Risk of bias in individual studies | 12 | Describe methods used for assessing risk of bias of individual studies (including specification of whether this was done at the study or outcome level), and how this information is to be used in any data synthesis. | 7 |
| Summary measures | 13 | State the principal summary measures (e.g., risk ratio, difference in means). | 7 |
| Synthesis of results | 14 | Describe the methods of handling data and combining results of studies, if done, including measures of consistency (e.g., I^2^) for each meta-analysis. | 7-8 |

| Risk of bias across studies | 15 | Specify any assessment of risk of bias that may affect the cumulative evidence (e.g., publication bias, selective reporting within studies). | 8 |
| --- | --- | --- | --- |
| Additional analyses | 16 | Describe methods of additional analyses (e.g., sensitivity or subgroup analyses, meta-regression), if done, indicating which were pre-specified. | 8-9 |
| **RESULTS** | | |  |
| Study selection | 17 | Give numbers of studies screened, assessed for eligibility, and included in the review, with reasons for exclusions at each stage, ideally with a flow diagram. | 10  Figure 1 |
| Study characteristics | 18 | For each study, present characteristics for which data were extracted (e.g., study size, PICOS, follow-up period) and provide the citations. | 10  Table 1 |
| Risk of bias within studies | 19 | Present data on risk of bias of each study and, if available, any outcome level assessment (see item 12). | 11 |
| Results of individual studies | 20 | For all outcomes considered (benefits or harms), present, for each study: (a) simple summary data for each intervention group (b) effect estimates and confidence intervals, ideally with a forest plot. | 11-12 |
| Synthesis of results | 21 | Present results of each meta-analysis done, including confidence intervals and measures of consistency. | 11-12 |
| Risk of bias across studies | 22 | Present results of any assessment of risk of bias across studies (see Item 15). | Appendix 4 |
| Additional analysis | 23 | Give results of additional analyses, if done (e.g., sensitivity or subgroup analyses, meta-regression [see Item 16]). | 11-12 |
| **DISCUSSION** | | |  |
| Summary of evidence | 24 | Summarize the main findings including the strength of evidence for each main outcome; consider their relevance to key groups (e.g., healthcare providers, users, and policy makers). | 12-16 |
| Limitations | 25 | Discuss limitations at study and outcome level (e.g., risk of bias), and at review-level (e.g., incomplete retrieval of identified research, reporting bias). | 17 |
| Conclusions | 26 | Provide a general interpretation of the results in the context of other evidence, and implications for future research. | 18 |
| **FUNDING** | | |  |
| Funding | 27 | Describe sources of funding for the systematic review and other support (e.g., supply of data); role of funders for the systematic review. | 19 |

**Additional file 2**

**Search Strategy : (Database: PubMed Embase Cochrane library ; Search completed 15th Jan 2022)**

--------------------------------------------------------------------------------------------------------------------------------------------------------------------------------------------------------

**PubMed**

1.

(("Critical Care"[Mesh]) OR ((((critical care[Title/Abstract]) OR (critically ill[Title/Abstract])) OR (intensive care[Title/Abstract])) OR (((((((((((((((Critical Illness[Title/Abstract]) OR (Critical Care[Title/Abstract])) OR (intensive care units[Title/Abstract])) OR (Burn units[Title/Abstract])) OR (coronary care units[Title/Abstract])) OR (respiration, artificial[Title/Abstract])) ) OR (ventilators, mechanical[Title/Abstract])) OR (pulmonary ventilation[Title/Abstract])) OR (respiratory insufficiency[Title/Abstract])) OR (multiple organ failure[Title/Abstract])) OR (systemic inflammatory response syndrome[Title/Abstract])) OR (respiratory distress syndrome, adult[Title/Abstract])) OR (sepsis[Title/Abstract])) OR (shock, septic[Title/Abstract]))))

2.

"Hyperphosphatemia"[Title/Abstract] OR "Hyperphosphatemia"[MeSH Terms]

3.

("Critical Care"[MeSH Terms] OR ("Critical Care"[Title/Abstract] OR "critically ill"[Title/Abstract] OR "intensive care"[Title/Abstract] OR ("critical illness"[Title/Abstract] OR "Critical Care"[Title/Abstract] OR "intensive care units"[Title/Abstract] OR "burn units"[Title/Abstract] OR "coronary care units"[Title/Abstract] OR "respiration artificial"[Title/Abstract] OR "ventilators mechanical"[Title/Abstract] OR "pulmonary ventilation"[Title/Abstract] OR "respiratory insufficiency"[Title/Abstract] OR "multiple organ failure"[Title/Abstract] OR "systemic inflammatory response syndrome"[Title/Abstract] OR "respiratory distress syndrome adult"[Title/Abstract] OR "sepsis"[Title/Abstract] OR "shock septic"[Title/Abstract]))) AND ("Hyperphosphatemia"[Title/Abstract] OR "Hyperphosphatemia"[MeSH Terms])

**Embase**

No. Query

#16. #3 AND #15

#15. #4 OR #5 OR #6 OR #7 OR #8 OR #9 OR #10 OR #11 OR

#12 OR #13 OR #14

#14. 'intensive care'/exp

#13. 'bacteremia':ab,ti AND [embase]/lim

#12. 'septic shock':ab,ti AND [embase]/lim

#11. 'sepsis':ab,ti AND [embase]/lim

#10. 'wound':ab,ti AND [embase]/lim

#9. 'trauma':ab,ti AND [embase]/lim

#8. 'icu':ab,ti AND [embase]/lim

#7. 'critical illness':ab,ti AND [embase]/lim

#6. 'intensive care':ab,ti AND [embase]/lim

#5. 'critically ill':ab,ti AND [embase]/lim

#4. 'critical care':ab,ti AND [embase]/lim

#3. #1 OR #2

#2. hyperphosphatemia:ab,ti AND ([embase]/lim OR

[medline]/lim)

#1. 'hyperphosphatemia'/exp

**Cochrane library**

ID Search

#1 ("intensive care"):ti,ab,kw (Word variations have been searched)

#2 ("critically ill"):ti,ab,kw (Word variations have been searched)

#3 ("critical care"):ti,ab,kw (Word variations have been searched)

#4 ("critical illness"):ti,ab,kw (Word variations have been searched)

#5 ("Burn"):ti,ab,kw (Word variations have been searched)

#6 ("acute respiratory distress syndrom"):ti,ab,kw (Word variations have been searched)

#7 ("truma"):ti,ab,kw (Word variations have been searched)

#8 ("septic shock"):ti,ab,kw (Word variations have been searched)

#9 #1 OR #2 OR #3 OR #4 OR #5 OR #6 OR #7 OR #8

#10 ("hyperphosphatemia"):ti,ab,kw(Word variations have been searched)

#11 #9 AND #10

#3|#1OR#2

**Additional file 3**

**Table S3:** **Studies needed for full-reviewed but not included in the current meta-analysis (n=7 trials).**

| No | Study | Reason of exclusion |
| --- | --- | --- |
| 1 | Barash Y, Klang E, Soffer S, Zimlichman E, Leibowitz A, Grossman E, Shlomai G. Normal-range emergency department serum phosphorus levels and all-cause mortality. Postgrad Med J. 2021 Feb;97(1144):83-88. | Reported without ICU admission |
| 2 | Haider DG, Lindner G, Wolzt M, Ahmad SS, Sauter T, Leichtle AB, Fiedler GM, Fuhrmann V, Exadaktylos AK. Hyperphosphatemia Is an Independent Risk Factor for Mortality in Critically Ill Patients: Results from a Cross-Sectional Study. PLoS One. 2015 Aug 7;10(8):e0133426. | Reported without ICU admission |
| 3 | Jang DH, Jo YH, Lee JH, Kim J, Park SM, Hwang JE, Lee DK, Park I, Lee CU, Lee SM. Moderate to severe hyperphosphataemia as an independent prognostic factor for 28-day mortality in adult patients with sepsis. Emerg Med J. 2020 Jun;37(6):355-361. | Reported without ICU admission |
| 4 | Tazmini K, Nymo SH, Louch WE, Ranhoff AH, Øie E. Electrolyte imbalances in an unselected population in an emergency department: A retrospective cohort study. PLoS One. 2019 Apr 25;14(4):e0215673. | Reported without ICU admission |
| 5 | Jung SY, Kim H, Park S, Jhee JH, Yun HR, Kim H, Kee YK, Yoon CY, Oh HJ, Chang TI, Park JT, Yoo TH, Kang SW, Lee H, Kim DK, Han SH. Electrolyte and mineral disturbances in septic acute kidney injury patients undergoing continuous renal replacement therapy. Medicine (Baltimore). 2016 Sep;95(36):e4542. | Repeated cohort study |
| 6 | Crowley KE, DeGrado JR, Charytan DM. Serum glucose and phosphorus concentrations during continuous renal replacement therapy using commercial replacement solutions with or without phosphorus. Hemodial Int. 2020 Jul;24(3):330-334. | Irrelevant to the current research |
| 7 | Kamr AM, Dembek KA, Reed SM, Slovis NM, Zaghawa AA, Rosol TJ, Toribio RE. Vitamin D Metabolites and Their Association with Calcium, Phosphorus, and PTH Concentrations, Severity of Illness, and Mortality in Hospitalized Equine Neonates. PLoS One. 2015 Jun 5;10(6):e0127684. | Irrelevant to the current research |
| 8 | Kim DW, Jung WJ, Lee DK, Lee KJ, Choi HJ. Association between the initial serum phosphate level and 30-day mortality in blunt trauma patients. J Trauma Acute Care Surg. 2021 Sep 1;91(3):507-513. |  |

**Additional file 4**

**Table S4 Summarized the adjustment for mortality reported by the included studies.**

|  | OR/HR (95% CI) | Adjustment |
| --- | --- | --- |
| Broman 2017 | aHR=1.20, 95 CI%: 1.00-1.5 | Study group, sex, age, total maximum SOFA score, minimum ionized calcium, **renal SOFA score** during the ICU stay and diagnosis class |
| Chen 2021 | aHR=2.04, 95 CI%: 1.91-2.30 | Age, gender, ethnicities, heart rate, MAP, respiratory rate, temperature, SaO2, score, SAPS ,OAS and ISS, disease types, lab variables, use of mechanical ventilation, **RRT**, or vasopressor on the first day. |
| Harbi 2021 | aOR=1.7, 95% CI: 1.1-2,29 | Age, APACHE II, sex, **serum creatinine** |
| Jung 2017 | aHR=1.05, 95% CI: 1.02-1.08 | Age, sex, and BMI at ICU admission, **urine output (2h)**. Charlson comorbidity index, SOFA score |
| Miller 2018 | aOR* | Site of infection |
| Rugg 2021 | aOR=3.96, 95% CI: 1.03–15.16 | Age, gender, pre-existing comorbidities (COPD, **chronic kidney disease**, etc.), ISS and admission phosphate levels |
| Sin 2021 | aOR=1.39, 95% CI: 1.15-1.68 | Phosphate level, admission diagnosis, gender, age, APACHE III, post cardiac arrest, treatment limitation, invasive ventilation |
| Wang 2021 | aOR=1.40, 95% CI: 1.16-1.70 | Age, male, ethnicity, ventilation ,vasopressin use, comorbidities (hypertension, **renal failure**, or congestive heart failure, etc.) laboratory data, SOFA |
| Kuo 2018 | aHR=2.15, 95% CI: 1.24-3.74 | Age, gender, surface area, inhalation Injury, **creatinine, urine output, AKI**, APACHE II, ABSI score |
| Suzuki 2013 | NA | NA |

*****aOR/HR = adjusted odds ratio/hazard ratio

**4. Additional file 5**

**Table S5 Summarizing conclusions of included studies concerning the hypophosphatemia and prognosis in critically ill patients.**

|  | Definition hypophosphatemia | Main results about the association between hypophosphatemia and prognosis in critically ill patients |
| --- | --- | --- |
| Broman 2017 | P< 0.7 mmol/L and no P values > 1.5 mmol/L] | Although we found that 180 days survival was significantly lower in hypophosphatemic patients, a Cox analysis adjusting for  confounding factors **did not find** **an increased risk** of death by increased hazard ratio. |
| Chen 2021 | P < 2.5 mg/L | After correction for potential confounders, hypophosphatemia at ICU admission **was not the independent risk factor** of 28 or 90-day mortality for general critically ill patients but was still a sign of worse clinical outcomes for ICU patients. |
| Harbi 2021 | less than 0.73 mmol/L | Our study showed that hyperphosphatemia, **but not hypophosphatemia**, during the first 24 h of ICU admission **was associated with an increase in-hospital mortality** in critically ill patients with sepsis or septic shock. |
| Jung 2017 | P < 2.5 mg/L | Furthermore, patients with increased phosphate levels during 24 h were at a higher risk of death **than those with stable or** **decreased phosphate levels** (P < 2.5 mg/L). (Table 4） |
| Miller 2018 | P < 2.5 mg/L | However, 28-day mortality was **not statistically significant** in those participants with time-weighted hypophosphatemia. |
| Rugg 2021 | P < 0.8 mmol/L | Logistic regression analysis **showed no association** between in-hospital mortality and phosphate levels <0.8 Mm， |
| Sin 2021 | P < 0.8 mmol/L | After using logistic regression to adjust for covariables, both hypophosphatemia and hyperphosphatemia at admission **remained independently associated with increased risk** of death in critically ill patients in Table 3. |
| Wang 2021 | P < 2.5 mg/L | Only hyperphosphatemia significantly correlated with in-hospital mortality, while the correlation between hypophosphatemia and in-hospital mortality **was not significant** (OR 0.91; 95% CI 0.70–1.19). |
| Kuo 2018 | NA | NA |
| Suzuki 2013 | P < 0.6 mmol/L | However, hypophosphatemic patients were also more severely ill and, after adjusting for other risk factors, hypophosphatemia **was not an independent risk factor** of mortality. |

**5.** **Additional file 6**

**Table S6: Quality assessment and overall risk of bias of included studies**

| First author / year | Patient selection | | | | Comparability | Outcome | | | Risk of bias |
| --- | --- | --- | --- | --- | --- | --- | --- | --- | --- |
|  | Representation of the exposed cohort | Selection of the non-exposed cohort | Ascertainment of exposure | Outcome of  interest not  present at start | Comparability of cohorts on the basis of the design or analysis | Assessment  of outcome | Was follow-up long enough for outcomes to occur | Adequacy of follow up of cohorts |  |
| Broman 2017 | ★ | ★ | ★ | ☆ | ★★ | ★ | ★ | ★ | 8 |
| Chen 2021 | ★ | ★ | ★ | ☆ | ★★ | ★ | ★ | ★ | 8 |
| Harbi 2021 | ★ | ★ | ★ | ☆ | ★★ | ★ | ★ | ★ | 8 |
| Jung 2017 | ★ | ★ | ★ | ☆ | ★★ | ★ | ★ | ★ | 8 |
| Miller 2018 | ★ | ★ | ★ | ☆ | ☆ | ★ | ★ | ★ | 6 |
| Rugg 2021 | ★ | ★ | ★ | ☆ | ★★ | ★ | ★ | ★ | 8 |
| Sin 2021 | ★ | ★ | ★ | ☆ | ★★ | ★ | ★ | ★ | 8 |
| Wang 2021 | ★ | ★ | ★ | ☆ | ★★ | ★ | ★ | ★ | 8 |
| Kuo 2018 | ★ | ★ | ★ | ☆ | ★★ | ★ | ★ | ★ | 8 |
| Suzuki 2013 | ★ | ★ | ★ | ☆ | ☆☆ | ★ | ★ | ★ | 6 |

**Abbreviations:** H=high quality; M=moderate quality; L= low quality.

**Note:** A study was given a maximum of one point in each item within the patient selection and outcome domains and given a maximum of two points for the Comparability domain with the following criteria:

1. **Representation of the exposed cohort**：Studies received 1 point if they recruited consecutive series of adult patients with blood phosphate concentration tested, or all included patients or did not miss a large number of patients.

2. **Selection of the non-exposed cohort**：Studies received 1 point if both groups of patients with or without hyperphosphatemia (defined by each author) were recruited from the same cohort.

3. **Ascertainment of exposure**: Studies received 1 point if they had been demonstrated to have abnormal blood phosphate concentration.

4. **Outcome of interest was not present at start of study**: Studies received points if they demonstrated the outcome of interest was not present at the start of the study.

5. **Comparability:** Studies received points if they controlled the disease severity (i.e., SOFA, SAPS3, ISS or APACHEII scores) (1 point); or any additional important factors such as age, gender or ethnicities, comorbidities, or there were no significant differences between hyperphosphatemia and normal phosphate concentration (1 point).

6. **Assessment of outcome**: Studies received 1 point if they had independent blind assessment or record linkage.

7. **Was follow-up long enough for outcomes to occur**: Studies received 1 point if they follow up until at least either inpatient mortality or for 30 days or had adequate record linkage.

8. **Adequacy of follow up for cohorts**: Studies received 1 point if all recruited subjects were all followed up, or the number lost to follow-up was unlikely to introduce bias (≤10%).

**7.** **Additional file 7**


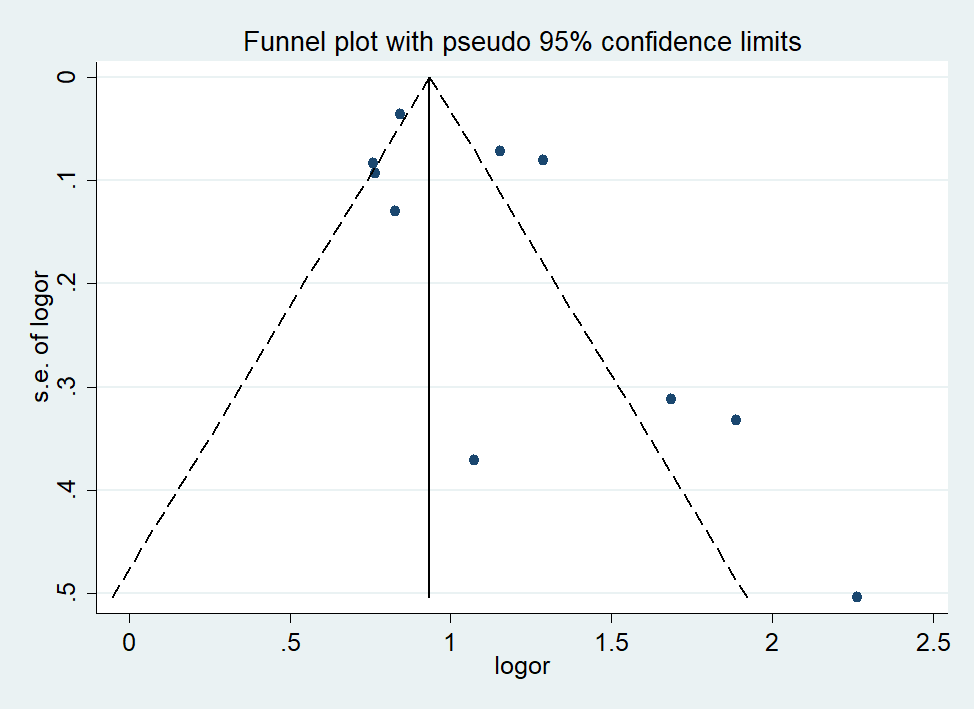


**Figure S7: Publication bias （outcome of between groups mortality, 10 studies）**
